# Increased Levels of Txa_2_ Induced by Dengue Virus Infection in IgM Positive Individuals Is Related to the Mild Symptoms of Dengue

**DOI:** 10.3390/v10030104

**Published:** 2018-02-28

**Authors:** Eneida S. Oliveira, Stella G. Colombarolli, Camila S. Nascimento, Izabella C. A. Batista, Jorge G. G. Ferreira, Daniele L. R. Alvarenga, Laís O. B. de Sousa, Rafael R. Assis, Marcele N. Rocha, Érica A. R. Alves, Carlos E. Calzavara-Silva

**Affiliations:** 1 Imunologia Celular e Molecular, Instituto René Rachou, Avenida Augusto de Lima, 1715, sala 406, Belo Horizonte 30190-002, Brazil; eneida.oliveira@minas.fiocruz.br (E.S.O.); stella.colombarolli@minas.fiocruz.br (S.G.C.); camila.nascimento@minas.fiocruz.br (C.S.N.); izabellaandrade@minas.fiocruz.br (I.C.A.B.); jorge.ferreira@minas.fiocruz.br (J.G.G.F.); daniele4.alvarenga@gmail.com (D.L.R.A.); rafael.assis@minas.fiocruz.br (R.R.A.); 2 Santa Casa de Misericórdia de Santo Antônio do Monte, Santo Antônio do Monte 35560-000, Brazil; lais.medicina2006@gmail.com; 3 Mosquitos Vetores: Endossimbiontes e Interação Patógeno-Vetor, Instituto René Rachou, Belo Horizonte 30190-002, Brazil; marcele@minas.fiocruz.br

**Keywords:** *Dengue virus*, eicosanoids, cyclooxygenase-2, 5-lipoxygenase, lipid bodies

## Abstract

The inflammatory process plays a major role in the prognosis of dengue. In this context, the eicosanoids may have considerable influence on the regulation of the *Dengue virus*-induced inflammatory process. To quantify the molecules involved in the cyclooxygenase and lipoxygenase pathways during *Dengue virus* infection, plasma levels of thromboxane A2, prostaglandin E2 and leukotriene B4; mRNA levels of thromboxane A2 synthase, prostaglandin E2 synthase, leukotriene A4 hydrolase, cyclooxygenase-2 and 5-lipoxygenase; and the levels of lipid bodies in peripheral blood leukocytes collected from IgM-positive and IgM-negative volunteers with mild dengue, and non-infected volunteers, were evaluated. *Dengue virus* infection increases the levels of thromboxane A2 in IgM-positive individuals as well as the amount of lipid bodies in monocytes in IgM-negative individuals. We suggest that increased levels of thromboxane A2 in IgM-positive individuals plays a protective role against the development of severe symptoms of dengue, such as vascular leakage.

## 1. Introduction

Dengue is an arthropod-borne viral disease caused by any of the four *Dengue virus* (DENV) serotypes (DENV-1 to -4). This virus belongs to the *Flaviviridae* family and is transmitted to humans through the bite of infected female mosquitoes of the genus *Aedes*. Dengue occurs in more than 128 countries in tropical and subtropical regions worldwide and is considered the most prevalent mosquito-borne viral disease affecting humans [1]. It is estimated that 3.9 billion people are at risk for DENV infection. In addition, an estimated 500,000 people with dengue require hospitalization each year, and approximately 2.5% of those affected will die [2].

In humans, after an incubation period of 4–8 days, DENV can induce a wide spectrum of illness, with most cases being asymptomatic or subclinical. Most patients recover after a self-limiting illness, while a small proportion of patients progresses to a severe disease state, which is primarily characterized by plasma leakage with or without bleeding [3]. The clinical course of dengue is influenced by multiple factors, such as DENV tropism and virulence, viral load, activation of the complement system, humoral and cellular immune response, as well as cytokines produced during DENV infection [4]. Furthermore, it is well known that the inflammatory process and the regulation thereof play major roles in the prognosis of dengue [5,6]. An exacerbated inflammatory response can trigger systemic tissue injury, leading to endothelial permeability, edema, inflammatory exudates and hematological abnormalities, such as leucopoenia and anemia [7]. In this context, eicosanoid molecules, which are bioactive signaling lipids, have considerable influence on the regulation of many inflammatory mechanisms involved in the pathogenesis of several diseases [8]. However, few studies have analyzed the role of eicosanoids during DENV infection [9,10,11].

In mammalian cells, eicosanoids can be synthesized within different cell compartments, including lipid bodies (LBs) [12]. These organelles are composed of a lipid core and a surface phospholipid monolayer. The core of the LB is composed of triglycerides, cholesterol esters and proteins, which are generally embedded in or adherent to the surface [13]. In the past, LBs were largely associated with lipid storage, but they are now recognized as dynamic and functionally active organelles involved in a variety of cellular functions, including lipid metabolism, cell signaling and inflammation [14]. LBs act as viral assembly platforms for several viruses, such as hepatitis C viruses, rotavirus, orthoreoviruses and DENV, participating primarily in viral encapsidation [15,16]. These organelles have been reported as the content of cytoplasmic vacuoles in macrophages [17], which have been observed the presence of DENV, facilitating viral replication [18].

The biosynthesis of eicosanoids depends on the availability of free arachidonic acid (AA). When tissues are exposed to diverse physiological and pathological stimuli, AA is produced from membrane phospholipids by phospholipase A_2_ and can then be converted by different enzymatic pathways into eicosanoids. In the cyclooxygenase (COX) pathway, free AA is converted into prostaglandin H_2_ (PGH_2_) by the action of the enzyme cyclooxygenase-1 or cyclooxygenase-2 (COX-2). Then, PGH_2_ is subsequently converted by cell-specific synthases into biologically active products, including prostaglandins and thromboxanes. In the lipoxygenase (LOX) pathway, AA can be converted into leukotrienes by different enzymes, including the enzyme 5-lipoxygenase (5-LOX) [8].

The eicosanoids of the COX pathway regulate a wide spectrum of processes. They elicit the cardinal signs of inflammation [19] and can also modulate the functions of neutrophils, monocytes, dendritic and natural killer cells, and T and B lymphocytes. In addition, some eicosanoids of the COX pathway induce vasoconstriction, activation of endothelial cells, and the activation and aggregation of platelets. On the other hand, the eicosanoids of the LOX pathway promote the recruitment of leukocytes, such as neutrophils, eosinophils, monocytes/macrophages, mastocytes, dendritic cells and effector T cells to sites of tissue damage [20]. These eicosanoids also induce the synthesis of pro-inflammatory cytokines by monocytes, as well as cytotoxicity mediated by NK cells and the differentiation of B-lymphocytes into IgE-producing cells [21].

Few studies have shown that eicosanoid-forming enzymes and their products are affected by DENV infection. Preeyasombat et al. (1999) [9] reported that the levels of thromboxane A_2_ (TXA_2_), a product of the COX pathway, were lower in the plasma from patients with dengue shock syndrome [9]. In another study, Loke et al. [10] demonstrated that the levels of leukotriene B_4_ (LTB_4_), a product of LOX pathway, were higher in the plasma from patients during the febrile and defervescence stages of dengue. These authors also observed increased levels of LTB_4_ in the neutrophil culture supernatant, which they attributed to the increased expression of *5-LOX* in these cells after in vitro DENV infection [10]. More recently, increased levels of prostaglandin E_2_ (PGE_2_), a product of the COX pathway, were detected in sera from patients during the febrile stage of dengue. Moreover, elevated *COX-2* gene expression in blood cells from dengue patients has also been observed [11]. However, to date, there is no study simultaneously comparing the COX and LOX pathways and their products during the natural course of dengue.

The aim of our study was to assess the plasma levels of TXA_2_ and PGE_2_ (products of the COX pathway) and LTB_4_ (product of the 5-LOX pathway). As LBs are organelles involved in the synthesis of eicosanoids, we also measured the level of eicosanoids in the peripheral blood leukocytes. In addition, we quantified the gene expression levels of *COX-2*, thromboxane A2 synthase (*TXA_2_S*) and prostaglandin E_2_ synthase (*PGE_2_S*), which are involved in the production of TXA_2_ and PGE_2_, and the mRNA levels of *5-LOX* and leukotriene A4 hydrolase (*LTA_4_H*), which are involved in the production of LTB_4_.

Here, we demonstrated that DENV infection may induce increased levels of TXA_2_ in IgM-positive dengue patients as well as an increased amount of LBs in the monocytes of IgM-negative dengue patients, suggesting that the balance of TXA_2_ and IgM levels plays a protective role against the development of severe symptoms of dengue, such as vascular leakage.

## 2. Materials and Methods

### 2.1. Ethical Aspects

This study was approved by the Ethics Committee from Instituto René Rachou/FIOCRUZ-MG (CAAE: 55135116.0.0000.5091, approved date: 18 August 2016). Participants involved in the study signed a written informed consent, and all analyzed data were anonymized.

### 2.2. Study Population

A cross-sectional study was conducted between March and May of 2017 in Santa Casa de Misericórdia de Santo Antônio do Monte, Santo Antônio do Monte city, Minas Gerais state, Brazil. Twenty-eight adult patients, above the age of 18, with a clinically characterized non-severe form of dengue infection were enrolled in the study. DENV infection was confirmed in the volunteers when at least one of the following tests was positive: (a) Dengue Virus NS1 ELISA (Euroimmun, Luebeck, Germany); (b) Anti-Dengue Virus ELISA (IgM) (Euroimmun); and (c) TRIOPLEX Dengue/Zika/ChikV kit (Eco Diagnóstica, Minas Gerais, Brazil). All dengue diagnostic tests were performed according to the manufacturer’s instructions. Dengue patients were subsequently divided into two study groups according to the absence or presence of anti-DENV IgM in their plasma. Twelve individuals (11 women and 1 men; median age = 33 years, range = 18.0 to 85.0) comprised the IgM-positive group. Sixteen individuals (12 women and 4 men; median age = 37.5 years, range = 18.0 to 61.0) comprised the IgM-negative group. The non-infected control group was composed of twelve individuals (6 women and 6 men; median age = 32.5 years, range = 27.0 to 56.0) with no signs or symptoms of dengue and with negative results in all dengue diagnostic tests.

### 2.3. Eicosanoids Quantification

Plasma obtained by the centrifugation of whole blood was stored at −80 °C. All samples were thawed only once, and the levels of eicosanoids were analyzed. Levels of TXA_2_ and PGE_2_ were indirectly assessed using the Thromboxane B_2_ ELISA Kit and Prostaglandin E Metabolite ELISA Kit, respectively. Levels of LTB_4_ were directly quantified using the Leukotriene B_4_ ELISA Kit. All reagents were purchased from Cayman Chemical (Ann Arbor, MI, USA), and all reactions were performed according to the manufacturer’s instructions.

### 2.4. Immunofluorescence Staining

Blood was centrifuged at 350× *g*, for 10 min at 18 °C. Pelleted blood cells were homogenized, and the number of leukocytes in each sample was counted in Turk’s solution (1:20) using a Neubauer chamber. An aliquot containing 5 × 10^5^ leukocytes was resuspended in 1% bovine serum albumin (Sigma-Aldrich, St. Louis, MO, USA) in 0.15 M PBS contained in 5-mL polypropylene round-bottom tubes (Becton Dickinson, Franklin Lakes, NJ, USA). Leukocytes were stained for 20 min at room temperature, protected from light, with anti-CD16 Alexafluor 700 (Clone 3G8), anti-CD56 PE-Cy5 (Clone B159), anti-CD3 PE (Clone UCHT1), anti-CD19 APC (Clone HIB19), anti-CD14 PerCP (Clone MψP-9), anti-CD45 V450 (Clone HI30), anti-CD4 APC (Clone RPA-T4) and anti-CD8 PerCP (Clone SK1), all purchased from BD Bioscience (Franklin Lakes, NJ, USA). Then, the leukocytes were stained for 20 min at room temperature with 5 µM Bodipy 493/503 (ThermoFisher Scientific, Waltham, MA, USA) in 0.15 M PBS. After staining, the erythrocytes were lysed, and leukocytes were washed. Cells were resuspended in 0.15 M PBS and stored in the refrigerator prior to acquisition on the flow cytometer within 24 h. Compensation was done using an Anti-Mouse Ig,k/Negative Control (FBS) Compensation Particles Set (BD Bioscience). Compensation parameters were calculated using the BD FACSDIVA 6.2 software (BD Bioscience). Data were acquired using the cytometer LSR Fortessa (BD Bioscience), and at least 200.000 events were acquired per sample. Data were analyzed using the FlowJo 10.1 software (FlowJo, Franklin Lakes, NJ, USA).

### 2.5. mRNA Quantification

Total RNA was extracted from peripheral blood leukocytes collected from DENV-infected individuals and healthy volunteers, using the PureLink™ RNA Mini Kit (ThermoFisher Scientific) following the manufacturer’s protocols. The purified RNA (50 ng) was reverse transcribed to cDNA using the GoScript Reserve Transcription System with random primers (Promega, Madison, WI, USA) according to the manufacturer’s instructions. The target genes were amplified and detected using TaqMan^®^ gene expression assays (ThermoFisher Scientific, cat. number 4,331,182-gene id: PTGS2–Hs00153133_m1, TBXAS1–Hs01022706_m1, PTGES2–Hs00228159_m1, ALOX5–Hs00167536_m1, LTA4H–Hs01075871 and ACTB–Hs_99999903_m1). Then, RNase H (2 U) (ThermoFisher Scientific) was added and the samples were incubated at 37 °C for 20 min. The RT-qPCR was performed using the StepOnePlus™ Real-Time PCR System (ThermoFisher Scientific). Reactions were performed in triplicate and included 5 μL of cDNA, 6.25 μM of each target TaqMan^®^ assay or human β-actin TaqMan^®^ assay used to normalize the gene expression data, 7.5 µL of TaqMan™ Gene Expression Master Mix (ThermoFisher Scientific) and water added to a final volume of 15 μL. Duplicates of non-template controls were included in each RT-qPCR experiment. The thermocycling conditions were as follows: initial holding (2 min at 50 °C and 10 min at 95 °C), followed by 40 cycles of 15 s at 95 °C and 1 min at 60 °C. The baseline and threshold for the cycle threshold (Ct) calculations were set automatically using the Sequence Detection Software, version 1.4 (Applied Biosystems, Waltham, MA, USA). The 2^−∆∆*C*t^ method was used to calculate the relative quantification [22,23], using the mean of all of the NI samples as the reference value.

### 2.6. Dengue Virus Load Quantification

Total nucleic acids were extracted from 240 μL of plasma that was centrifuged at 20,000× *g* for 1.5 h at 4 °C to concentrate the viral particles. After centrifugation, the supernatant was discarded and 50 μL of buffer containing Tris Base (Inlab, São Paulo, Brazil), EDTA (Synth, São Paulo, Brazil), NaCl (Synth) and proteinase K (Qiagen, Hilden, Germany) was added. All samples were subsequently processed in the Thermal Cycler Veriti (ThermoFisher Scientific), at 56 °C for 5 min and 98 °C for 15 min. Then, the obtained nucleic acids were used to determine the DENV viral load (number of DENV RNA copies per mL of plasma), which was quantified using a LightCycler^®^ 96 (Roche, Mannheim, Germany) and the following primers and probe: DENV-F: 5′-AAG GAC TAG AGG TTA GAG GAG ACC C-3′, DENV-R: 5′-CGT TCT GTG CCT GGA ATG ATG-3′ and DENV-Probe: 5′-/TEX615/AAC AGC ATA TTG ACG CTG GGA GAG ACC AGA/3IAbRQSp/3′ [24]. Reactions were performed in duplicate and included 2.5 µL of nucleic acids, 0.25 µL of each primer, 0.1 µL of probe, 2.5 µL of TaqMan Fast Virus 1-Step Master Mix (ThermoFisher Scientific) and water added to a final volume of 10 µL. Duplicates of non-template controls were included in the RT-qPCR assays. Thermocycling conditions were as follows: initial holding (20 s at 95 °C) followed by 40 cycles of 3 s at 95 °C and 30 s at 60 °C. For quantification purposes, the sequences of the DENV amplicons (3′ UTR), were cloned (pGEMT-Easy plasmid, Promega), amplified and then serially diluted to generate a standard curve, as previously described by Richardson et al. (2006) [25].

### 2.7. Analyses of Vacuolated Monocytes

The morphology of the monocytes was analyzed using blood smears stained with a hematology staining kit (Instant-Prov, Newprov, Paraná, Brazil), according to the manufacturer’s instructions. The percentage of vacuolated monocytes was determined by counting one hundred cells using light microscopy at 1000× magnification.

### 2.8. Statistical Analysis

The D’Agostino and Pearson omnibus test was used to evaluate the normality of data distribution. ANOVA, followed by the Student Newman–Keuls method, was used to compare multiple normal samples. The Kruskal–Wallis test, followed by the Dunn’s method, was used to compare multiple non-normal samples. The unpaired nonparametric Mann–Whitney *t*-test was used to evaluate the data of viral load. The Prism 6^®^ software package (GraphPad Software, San Diego, CA, USA) was used for statistical tests. Differences were considered significant when a *p* value ≤ 0.05 was obtained.

## 3. Results

### 3.1. Dengue Diagnosis

Out of 40 volunteers enrolled in this study, 12 healthy volunteers were considered non-infected by DENV, based upon negative results from RT-qPCR, ELISA NS1 and ELISA IgM tests. The 28 remaining volunteers presented dengue symptoms and were positive in at least one of the three tests used (17 were positive based upon RT-qPCR, 20 were positive based upon ELISA NS1 and 16 were positive based upon ELISA IgM). Thus, 28 volunteers were considered as DENV-infected subjects (Table 1). Due to the wide variation in days of symptoms reported by the volunteers (1–12 days of symptoms), the volunteers were divided into two groups according to the presence or absence of IgM.

### 3.2. Plasma from IgM-Positive Individuals Has Increased Levels of TXA_2_

**Figure 1:**
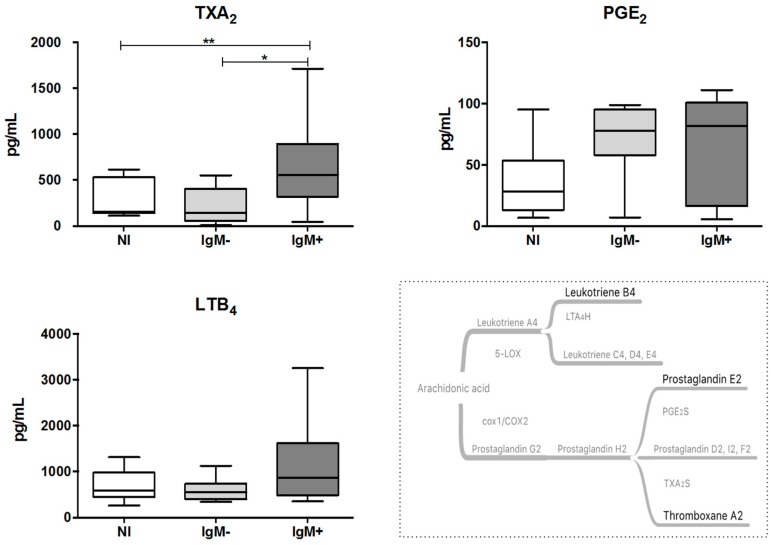
Quantification of eicosanoid TXA_2_, PGE_2_ and LTB_4_ in plasma from dengue-infected individuals. The eicosanoids were quantified in plasma samples using commercial ELISA kits. Data represent the median, quartiles and extreme values. NI = Non-infected individuals (*n* = 12). IgM^−^ = DENV-infected individuals showing the absence of IgM in plasma (*n* = 12). IgM^+^ = DENV-infected individuals showing the presence of IgM in plasma (*n* = 1). (*) *p* < 0.05 and (**) *p* < 0.01, TXA_2_ and LTB_4_ were determined by ANOVA/Student Newman–Keuls test, and PGE_2_ was determined by the Kruskal–Wallis/Dunn’s method.

To evaluate the impact of DENV infection on the production of eicosanoids, we assessed the levels of TXA_2_ and PGE_2_, by measuring their stable metabolites, and LTB4 in the plasma from DENV-infected individuals. As shown in Figure 1, the IgM-positive group displayed higher plasma levels of TXA_2_ compared with the non-infected group (555.9 pg/mL versus 155.3 pg/mL; *p* < 0.05) and the IgM-negative group (555.9 pg/mL versus 141.3 pg/mL; *p* < 0.01). The plasma levels of PGE_2_ and LTB_4_ were not significantly altered by the DENV infection.

### 3.3. Monocytes from IgM-Negative Individuals Displayed Increased Levels of LBs

**Figure 2:**
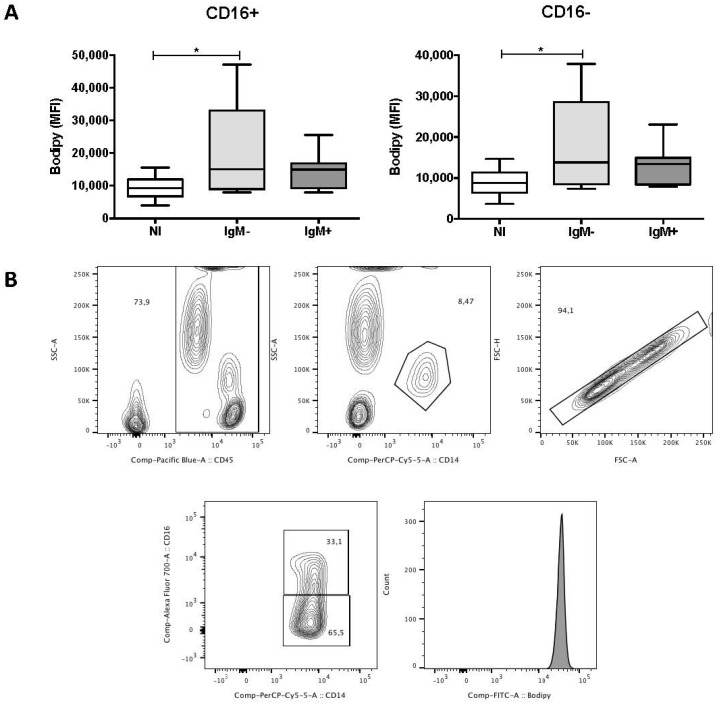
Lipid bodies in subpopulations of monocytes of dengue patients. (**A**) Effect of DENV infection on the median fluorescence intensities (MFI) of Bodipy 493/503 in CD16^+^ and CD16^−^ monocytes, analyzed by flow cytometry. The antibodies used were anti-CD16 Alexafluor 700 (Clone 3G8), anti-CD14 PerCP (Clone MψP-9), and anti-CD45 V450 (Clone HI30). Data represent the median, quartiles and extreme values. NI, Non-infected individuals (*n* = 8); IgM^−^, DENV-infected individuals showing the absence of IgM in plasma (*n* = 10). IgM^+^, DENV-infected individuals showing the presence of IgM in plasma (*n* = 10). (*) *p* < 0.05. LB in both subpopulations of monocytes was determined by ANOVA/Student Newman–Keuls test; (**B**) gating strategy used in the flow cytometry analysis of LBs in subpopulations of monocytes from a dengue patient. Leukocytes were gated on a SSC-A versus CD45 plot. Monocytes were then further gated on a SSC-A versus CD14 plot. Next, single cells were gated on a FSC-H versus FSC-A plot. After that, CD16^+^ and CD16^−^ monocytes were gated on a CD14 versus CD16 plot. Histograms were used to obtain the median fluorescence intensity (MFI) of Bodipy 493/503.

To investigate the effect of DENV infection on the levels of LBs in the peripheral blood leukocytes, we measured the median fluorescence intensity (MFI) of Bodipy 493/503, a green fluorescent dye that binds to the neutral lipids in LBs. Figure 2A shows that the MFI of Bodipy 493/503 in CD16^+^ (15,007 versus 9238) and CD16^−^ (13,824 versus 8806) monocytes from the IgM-negative group was higher compared with the non-infected group (*p* < 0.05). In contrast, there was no difference in the MFI of Bodipy 493/503 when comparing subpopulations of monocytes from the non-infected and IgM-negative groups. The gating strategy used to analyze the monocytes is demonstrated in Figure 2B. DENV infection did not alter the MFI of Bodipy 493/503 in neutrophils, eosinophils, lymphocytes, NK and NK T cells [26].

### 3.4. Expression Levels of COX-2 and TXA_2_S Genes Were Lower in Leukocytes from IgM-Positive Individuals

**Figure 3:**
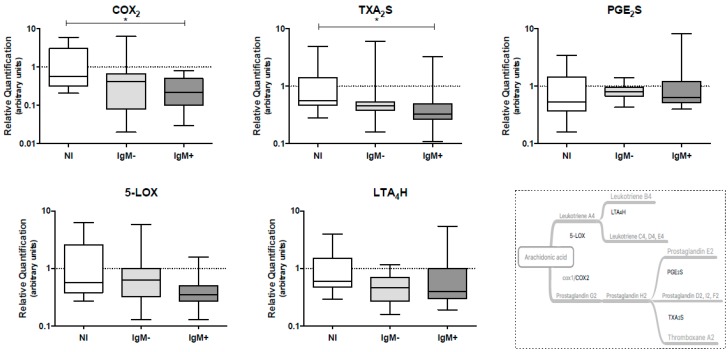
Quantification of mRNA levels coding for the eicosanoid-forming enzymes COX_2_, TXA_2_S, PGE_2_S, 5-LOX and LTA_4_H in peripheral blood leukocytes from dengue patients. The quantification of eicosanoid-forming enzymes was performed by RT-qPCR, using TaqMan^®^ gene expression assays and human β-actin TaqMan^®^ assay used to normalize the gene expression. Data represent the median, quartiles and extreme values. NI, Non-infected individuals (*n* = 12); IgM^−^, DENV-infected individuals showing the absence of IgM in plasma (*n* = 12); IgM^+^, DENV-infected individuals showing the presence of IgM in plasma (*n* = 15). (*) *p* < 0.05. All analyses were determined by the Kruskal–Wallis/Dunn’s method.

The effect of DENV infection on the mRNA levels of genes coding for eicosanoid-forming enzymes, such as *COX-2*, *TXA_2_S* and *PGE_2_S* (COX pathway), and *5-LOX* and *LTA_4_H* (5-LOX pathway), in peripheral blood leukocytes collected from DENV-infected individuals was assessed by qRT-PCR. We observed that the IgM-positive group presented lower levels of mRNA coding for the *COX-2* gene (0.22 versus 0.58; *p* < 0.05) and the *TXA_2_S* gene (0.33 versus 0.56; *p* < 0.05) when compared to the non-infected group. The gene expression levels of *PGE_2_S*, *5-LOX* and *LTA_4_H* were not altered by the DENV infection (Figure 3).

### 3.5. The Dengue Virus Load Was Higher in Plasma from IgM-Negative Patients

**Figure 4:**
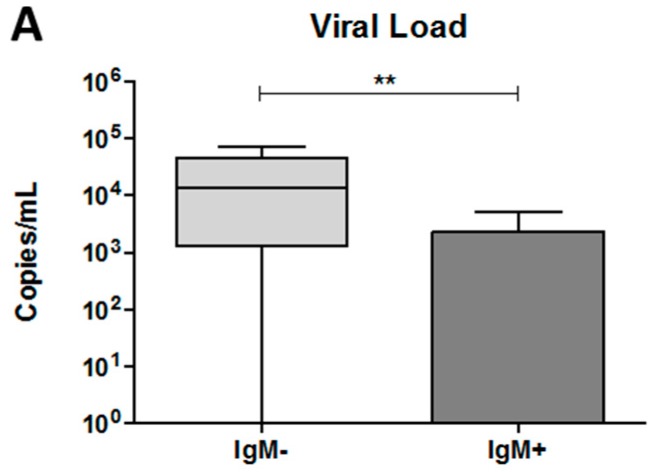

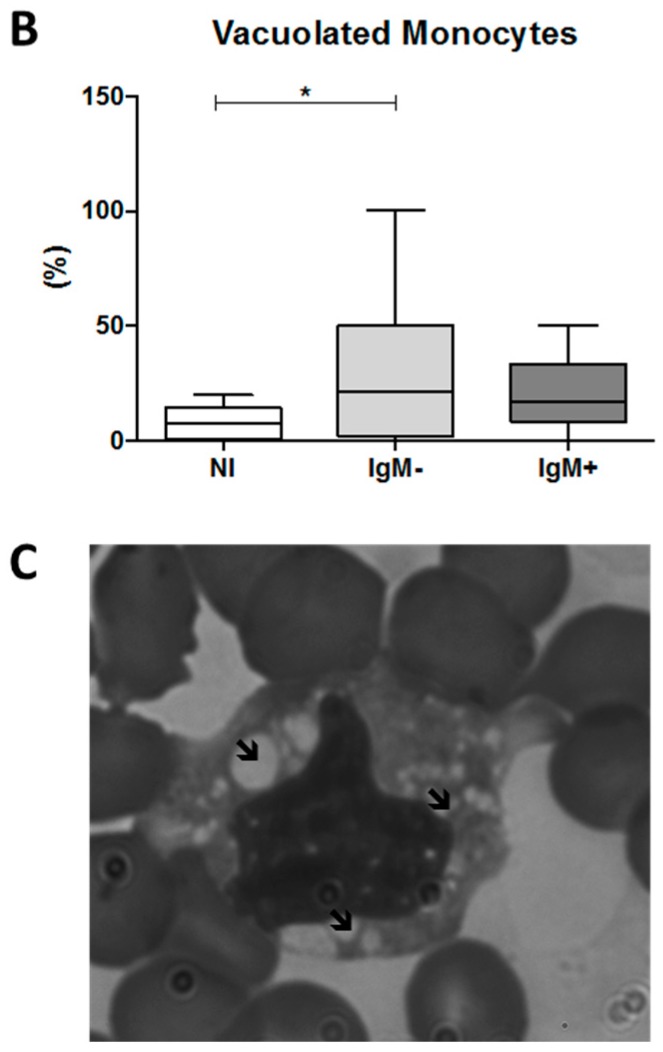
Quantification of DENV viral load and percentage of vacuolated monocytes in the blood collected from dengue patients. Data represent the median, quartiles and extreme values. (**A**) The quantification of viral load was performed by RT-qPCR. DENV RNA copies per mL of plasma. IgM^−^, DENV-infected individuals showing the absence of IgM in plasma (*n* = 12); IgM^+^, DENV-infected individuals showing the presence of IgM in plasma (*n* = 16). The analysis was determined by the Mann–Whitney *t*-test; (**B**) percentage of vacuolated monocytes was determined using blood smears. NI (*n* = 12), IgM^−^ (*n* = 12), IgM^+^ (*n* = 11). (*) *p* < 0.05 and (**) *p* < 0.01. The analysis was determined by ANOVA/Student Newman–Keuls test; (**C**) representative image of a vacuolated monocyte in the blood from a dengue patient, 1000× magnification. ↙ = indicate the vacuoles.

To determine the viral load in the plasma of each dengue patient, we performed qRT-PCR. As shown in Figure 4A, an increase in the number of viral copies was observed in the IgM-negative group compared with the IgM-positive group (13,335 copies /mL versus 0.0 copies/mL; *p* < 0.001).

### 3.6. An Increased Percentage of Vacuolated Monocytes Was Observed in IgM-Negative Individuals

To investigate whether DENV induces cytoplasmic vacuolization in monocytes, we assessed the percentage of vacuolated monocytes in blood smears from dengue patients. We observed that the IgM-negative group presented a percentage of vacuolated monocytes significantly higher than that of the non-infected group (21.6% versus 7.2%; *p* < 0.05, Figure 4B). An example of a vacuolated monocyte in the blood from a DENV-infected individual is presented in Figure 4C.

## 4. Discussion

In this work, we aimed to evaluate whether DENV infection affects the synthesis of eicosanoids mediated by the COX and 5-LOX enzymes. Thus, the levels of TXA_2_, PGE_2_ and LTB_4_, as well as the levels of mRNA coding for the *TXA_2_S, PGE_2_S* and *LTA_4_H*, *COX-2* and *5-LOX* genes were assessed in blood collected from 28 DENV-infected individuals with mild symptoms and from non-infected volunteers. Based on the chronology of the humoral response to dengue [1,27], the infected individuals were subdivided into the IgM-negative group and IgM-positive group, representing the early and late stages of the acute phase of dengue, respectively.

Our data revealed increased levels of TXA_2_ in IgM-positive dengue patients. Indeed, TXA_2_ is one of the most potent eicosanoids involved in vasoconstriction and platelet aggregation [28]. Therefore, the increased levels of TXA_2_, associated with unaltered levels of the vasodilators PGE_2_ and LTB_4_, may have contributed to the absence of severe symptoms of dengue, such as vascular extravasation, in the individuals involved in our study. In fact, it has been suggested that TXA_2_ may play a protective role in dengue, since decreased levels of TXA_2_ in the blood of patients with dengue have been associated with the development of hypovolemic shock signals [9]. Moreover, PGE_2_ seems to play a deleterious role in dengue prognosis, since increased plasma levels of PGE_2_ were observed in a group of 11 individuals with dengue who developed vascular extravasation, as reported by Lin et al. (2017) [11].

PGE_2_ and TXA_2_ are synthesized from AA metabolism by the action of two cyclooxygenase isoforms, known as cyclooxygenase-1 (with constitutive expression) and COX-2 (inducible), to generate PGH_2_. Then, PGH_2_ can be converted by the enzymes PGE_2_S or TXA_2_S into PGE_2_ and TXA_2_, respectively. TXA_2_ has a short half-life and is quickly converted to the stable but physiologically inactive metabolite thromboxane B_2_ [20]. The production of LTB_4_ begins when AA is metabolized by 5-LOX to form an unstable epoxy intermediate, which undergoes hydrolysis to generate leukotriene A_4_. Then, leukotriene A_4_ is converted to LTB_4_ by the action of the enzyme LTA_4_H [29]. TXA_2_ [30], PGE_2_, [31] and LTB_4_ [32] can be produced by various cells, including leukocytes. Therefore, to verify whether leukocytes were involved with the increased levels of TXA_2_ observed after DENV infection and IgM production, we first quantified the LBs in the main peripheral blood leukocyte populations, since these organelles are the main sites of intracellular eicosanoid production [14].

We observed that the subpopulations of CD16^−^ and CD16^+^ monocytes of the IgM-negative group displayed higher levels of LBs compared with the non-infected group, whereas the other leucocytes presented levels of LBs similar to those of the uninfected group, independent of presence or absence of IgM [26]. In fact, Samsa et al. (2009) [33] observed that BHK cells presented an increased number of LBs after in vitro infection by DENV-2. The authors postulated that the LBs sequester DENV capsid proteins and participate in the encapsidation of the DENV genome [33]. In addition, Carvalho et al., (2012) [16] confirmed the interaction between viral capsid protein and the protein perilipin 3 (TIP47) from LBs [16]. Moreover, Assunção-Miranda et al. (2010) [34] demonstrated that the peripheral blood leukocytes from dengue patients displayed increased amounts of LBs. However, in their study, the specific population of leukocytes that showed the increase of these organelles was not discriminated [34]. Our study, therefore, is the first to identify monocytes as the leukocytes that display altered levels of LBs in patients infected by DENV.

Thus, in this study, we suggest that the increase of the LB levels observed in monocytes of the IgM-negative group, despite not being associated with alterations in the plasma levels of eicosanoids or the mRNA levels of genes involved in eicosanoid-forming enzymes in leukocytes, is due to DENV infection and replication, since this increase was observed in the subpopulations of both positive and negative CD16 monocytes, both of which are permissive cells to viral infection and replication [35]. Our hypothesis is supported by the fact that the IgM-negative dengue patients also presented high DENV loads in the bloodstream. Moreover, the IgM-negative group presented a higher percentage of vacuolated monocytes in their blood, compared with the IgM-positive group. Corroborating our results, Mosquera et al. (2005) [18] also observed high vacuolization in human monocytes after in vitro infection with DENV-2 [18].

Because gene expression levels of *COX-2* and *TXA_2_S*, which are involved in TXA_2_ production, were lower in the IgM-positive group, we hypothesize that other sources of TXA_2_, such as platelets and/or vascular endothelial cells, both of which are activated after DENV infection [36,37], may have contributed to the elevated TXA_2_ plasma levels of the IgM-positive group. In fact, platelets are the major source of TXA_2_ in vivo [30], and it has been observed that endothelial cells are also able to synthesize TXA_2_ [38]. In this context, a recent study conducted by Lin et al. (2017) [11] reported an increase in *COX-2* mRNA levels in whole blood of dengue patients who developed severe illness, whereas the patients involved in our study presented only mild symptoms of dengue [11]. Red blood cells and platelets may also express *COX-2* [39,40], which may have contributed to the increased levels of *COX-2* mRNA observed by the authors. Moreover, an in vitro study conducted by Loke et al. [10] in 2013 showed an increase in 5-LOX expression in neutrophils stimulated by DENV-2, which was followed by LTB_4_ production [10]. This contrasting result can be explained by the short time of exposure (7 h) and high viral load used in their study, which does not reflect the natural infection and, consequently, the progression of the disease or the immune response. Our work is the first that describes, ex vivo, the influence of DENV infection on the levels of *TXA_2_S*, *PGE_2_S* and *LTA_4_H* mRNA.

**Figure 5:**
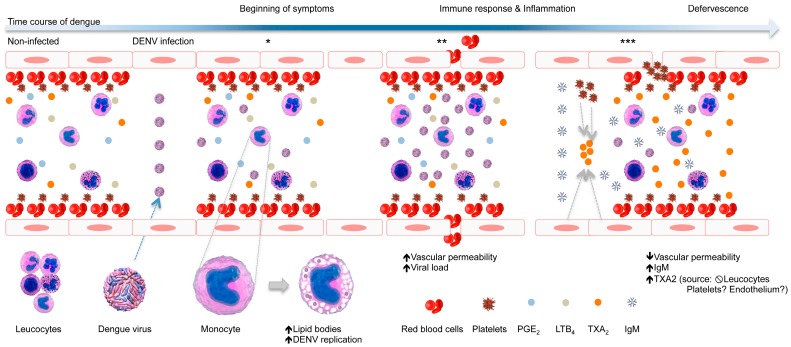
Schematic model of the role of LB and TXA_2_ in dengue prognosis. During the time course of DENV infection, the virus triggers morphological and functional changes in monocytes, increasing the amount of LBs and vacuoles, which may be optimal sites of viral replication (*). Further, a high viral load and an unbalanced immune response could lead to the onset of dengue symptoms, including vascular damage with plasma leakage and bleeding (**). However, after seroconversion, characterized by the production of IgM, high levels of TXA_2_ are produced, putatively by platelets and/or endothelial cells, which may trigger vasoconstriction and platelet aggregation, preventing the intense vascular leakage and bleeding observed in severe dengue cases (***).

Here, we propose a model for the role of LB and TXA_2_ in dengue prognosis, as presented in Figure 5. During the time course of DENV infection, the virus triggers morphological and functional changes in monocytes, increasing the amount of LBs and vacuoles therein, which may be optimal sites of viral replication. Further, a high viral load and an unbalanced immune response could lead to the onset of dengue symptoms, including vascular damage with plasma leakage and bleeding. However, after seroconversion, characterized by the production of IgM, a high amount of TXA_2_ is produced, putatively by platelets and/or endothelial cells, which induces vasoconstriction and platelet aggregation, thereby preventing the intense vascular leakage and bleeding observed in severe dengue cases.

Taken together, our data led us to infer that during the early stages of the acute phase of dengue, when any or low levels of IgM can be detected, there is an increase in the levels of LBs in monocytes. Given that these cells are susceptible to DENV infection and the time course of infection coincides with the highest viral load but no concomitant changes were observed in the levels of mRNA coding for the enzymes involved in eicosanoids synthesis, we hypothesized that in the early stage of the disease, LBs are related to the replication of DENV in monocytes. We also concluded that the activation of the eicosanoid synthesis pathways occurs exclusively at the beginning of the defervescence of the disease, characterized by the production of TXA_2_. Since the individuals analyzed in our study did not develop severe dengue and TXA_2_ is a potent vasoconstrictor and platelet activator, we believe that this lipid mediator may play an important role in limiting the development of vascular leakage during DENV infection. More studies are warranted to investigate the role of LBs as well as the COX- and 5-LOX-mediated eicosanoid synthesis pathways during DENV infection. Finally, our data indicate that LBs could be interesting therapeutic targets to control viral replication during DENV infection and that eicosanoids may serve as important biomarkers of dengue prognosis.

## Figures and Tables

**Table 1:** Profile of molecular and serological tests of volunteers.

Volunteer	RT-qPCR	ELISA NS1	ELISA IgM
1	+	+	+
2	+	−	−
3	−	−	+
4	+	+	+
5	+	+	−
6	−	−	+
7	−	−	+
8	+	+	−
9	+	+	−
10	−	+	−
11	−	+	+
12	+	+	−
13	−	+	+
14	−	+	−
15	−	+	+
16	+	+	+
17	−	+	+
18	+	+	+
19	+	+	−
20	+	+	−
21	+	−	+
22	−	−	+
23	+	+	−
24	+	+	−
25	+	+	−
26	−	−	+
27	+	+	+
28	+	−	+
29	−	−	−
30	−	−	−
31	−	−	−
32	−	−	−
33	−	−	−
34	−	−	−
35	−	−	−
36	−	−	−
37	−	−	−
38	−	−	−
39	−	−	−
40	−	−	−

+ and − = Positive or negative result at the given test, respectively.

## Abbreviations

COX	Cyclooxygenase
LOX	Lipoxygenase
TXA_2_	thromboxane A2
PGE_2_	prostaglandin E2
LTB_4_	leukotriene B4
*TXA_2_S*	thromboxane A2 synthase
*PGE_2_S*	prostaglandin E2 synthase
*LTA_4_H*	leukotriene A4 hydrolase
*COX-2*	cyclooxygenase-2
*5-LOX*	5-lipoxygenase
DENV	Dengue virus
LB	lipid bodies
AA	arachidonic acid
PGH_2_	prostaglandin H2
MFI	median fluorescence intensity
N	Non-infected
